# Global microvascular ischaemia following Takotsubo cardiomyopathy with left ventricular function recovery

**DOI:** 10.1093/ehjcr/ytab093

**Published:** 2021-03-10

**Authors:** Amrit Chowdhary, Sharmaine Thirunavukarasu, Nick Jex, Eylem Levelt

**Affiliations:** Division of Biomedical Imaging, Leeds Institute of Cardiovascular and Metabolic Medicine (LICAMM), University of Leeds, Worsley Building, Leeds LS2 9JT, UK

An 86-year-old woman presented to hospital with worsening dyspnoea over the past 7 days. An admission 12-lead electrocardiogram showed ST-elevation with T-wave inversion in leads V2–V6, I, II, III, and aVF (*Figure 1A*). Blood tests showed an elevated troponin I of 10 612 ng/L (<57 ng/L) and N-terminal pro B-type natriuretic peptide of 34 811 ng/L (<34 ng/L). Transthoracic echocardiogram demonstrated left ventricular ejection fraction of 35% with akinesis of the mid-anterior, mid-septal, and all apical segments (*Figure 1B* and *C*). A diagnosis of late anterior ST-elevation myocardial infarction was made and given the low likelihood of clinical benefit with revascularization, she was medically managed.

**Figure 1:**
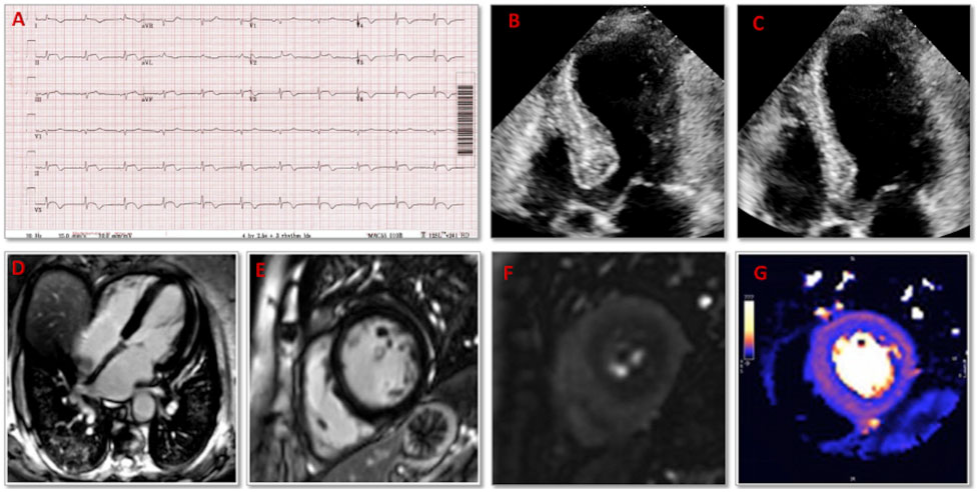
(*A*) An admission 12-lead electrocardiogram showing ST-elevation with T-wave inversion across leads V2–V6, I, II, III, and aVF. (*B*) End-diastolic four-chamber transthoracic echocardiogram view at admission. (*C*) End-systolic four-chamber transthoracic echocardiogram view at admission. (*D*) Four-chamber cardiovascular magnetic resonance late-gadolinium enhanced left ventricular mid-slice showing absence of fibrosis or myocardial infarction. (*E*) Short-axis cardiovascular magnetic resonance late-gadolinium enhanced left ventricular mid-slice showing absence of fibrosis in the area of the perfusion defect. (*F*) Basal left ventricular short-axis cardiovascular magnetic resonance perfusion slice showing marked subendocardial circumferential perfusion defect, with (*G*) corresponding cardiovascular magnetic resonance quantitative perfusion map demonstrating subendocardial circumferential hypoperfusion.

At 8-week outpatient review, she reported worsening dyspnoea and atypical chest pain. An adenosine stress cardiovascular magnetic resonance scan (CMR) was performed which revealed normal bi-ventricular size and function, no regional wall motion abnormalities, and no myocardial scar on late gadolinium-enhanced imaging (*Figure 1D* and *E*). Adenosine stress perfusion imaging demonstrated marked circumferential perfusion defects with a globally reduced myocardial blood flow at stress of 1.21 mL/g/min (*Figure 1E* and *F*). Subsequently, an invasive coronary angiogram was performed, which revealed mild–moderate stenosis in the left anterior descending artery only. In the absence of significant epicardial coronary artery stenosis, the perfusion defects on CMR may be due to coronary microvascular dysfunction (CMD).

Retrospectively, a diagnosis of Takotsubo cardiomyopathy with CMD was made. Supporting this, the patient with hindsight reported a preceding stressor: her wallet being stolen on the day of admission.

## Supplementary material

Supplementary material is available at *European Heart Journal - Case Reports* online.

**Slide sets:** A fully edited slide set detailing this case and suitable for local presentation is available online as Supplementary data.

**Consent:** The authors confirm that written consent for submission and publication of this case report including images and associated text has been obtained from the patient in line with COPE guidance.

**Conflict of interest:** None declared.

**Funding:** None declared.

